# Core-Shell-Structured Particle Reinforced A356 Matrix Composite Prepared by Powder-Thixoforming: Effect of Reheating Temperature

**DOI:** 10.3390/ma11091718

**Published:** 2018-09-13

**Authors:** Tijun Chen, Libo Geng, He Qin, Min Gao

**Affiliations:** State Key Laboratory of Advanced Processing and Recycling of Nonferrous Metals, Lanzhou University of Technology, Lanzhou 730050, China; 18298372557@163.com (L.G.); qinhe19900927@126.com (H.Q.); gaom199409@163.com (M.G.)

**Keywords:** Core-shell-structured reinforcing particle, Al matrix composite, powder thixoforming, toughening mechanism, strengthening model

## Abstract

A novel core-shell-structured Ti-(Al-Si-Ti) particle (Ti-(Al-Si-Ti)_p_) reinforced A356 matrix composite was fabricated by a new method, powder thixoforming, which combines the merits of both powder metallurgy and semisolid thixoforming. The effects of reheating temperature on the microstructure and tensile properties of the resulting composite were investigated. The results indicated that the thickening of the Al-Si-Ti compound shells, with rising the reheating temperature, significantly enhanced the strengthening role, but the fracture and peeling of the shells, at higher than 600 °C, impaired the strengthening effect. The composite formed at 600 °C had a favorable tensile elongation of 8.3% besides high tensile strengths. During tensile testing, the Ti@(Al-Si-Ti)_p_ frequently fractured across the Ti cores and occasionally cracked around the Ti cores, but preferentially fractured between the outer cracked shells and the inner cores for the composites thixoformed at higher than 600 °C. The delayed formation of cracks in the Ti-(Al-Si-Ti)_p_ and the small size of the cracks contributed to ductility improvement. The MSL model, modified according to the Ti@(Al-Si-Ti)_p_ characteristics, was essentially suitable for predicting the yield strength of such composites. The largest contribution to the strength was resulted from solid solution strengthening of Ti element, but the strengthening role from geometrically necessary dislocations was significantly improved as the reheating temperature rose from 590 °C to 600 °C.

## 1. Introduction

Particle reinforced Al matrix composites (PRAMCs) have large application potential in the fields of aerospace, weapon, automobile and other high-end equipment because of their high specific strength and stiffness [1]. The most commonly used reinforcements are some ceramic particles. But the mechanical properties of the resulting PRAMCs are always relatively low due to weak interfacial bonding strength resulting from poor interfacial wettability and possible interfacial reaction [2]. However, in-situ PRAMCs have a clean and strong bonding interface, and thus, high mechanical properties [3,4]. Al_3_Ti particles (Al_3_Ti_p_) have low density (3.4 g/cm^3^), high melting point (1340 °C), high Young’s modulus (216 GPa), and equivalent coefficient of thermal expansion (CTE) to Al matrix alloys, so they are considered one of the most promising in situ reinforcements for Al matrix composites [1,5,6].

However, the Al_3_Ti_p_/Al matrix composites, similar to other ceramic particle reinforced Al matrix composites, always have very poor elongation, although they possibly have high tensile strength [3,4,5]. The reason is that the Al_3_Ti_p_ are also quite brittle and easy to fracture. Cracks with the same size to the Al_3_Ti_p_ can instantaneously generate within the Al_3_Ti_p_ when tensile testing proceeds to a given degree, which then lead the composite to prematurely fracture, resulting in a poor tensile elongation. If the size of Al_3_Ti_p_ is significantly decreased, their fracture can be obviously delayed, and thus, the mechanical properties, especially the elongation, will be improved. For this purpose, post plastic processing is always carried out or high-intensity ultrasonic vibration is introduced during preparation by liquid casting [2,6,7,8]. But these technologies undoubtedly complicate the fabricating process and increase the cost of the composites.

Song et al. prepared a core-shell-structured particle reinforced Al matrix composite by powder metallurgy (PM) [9,10]. The reinforcements include a ductile Ti core and a hard Al_x_Fe_y_ compound shell (for convenience, this kind of reinforcements is presented as Ti@Al_x_Fe_yp_) that in situ formed during preparation. For this composite, the first formed cracks in the reinforcements should be only limited within the thin compound shells, and thus, the crack sizes are obviously smaller than those in monolithic Al_x_Fe_y_ particles with the same size to the core-shell-structured ones. In addition, two tips of each crack face the ductile Ti and Al matrix, respectively, so crack propagation can be delayed by blunting of the crack tips. The tensile elongation of the composite thereby is enhanced. However, the experimental results indicated that the tensile strain of the resulting composite was less than 1%. The reason is that there were numerous small voids in the composite due to the processing characteristics of PM. Afterwards, they prepared a Ti@Al_3_Ti_p_/Al matrix composite using the same technology, its tensile elongation was improved as compared with the Fe@Al_x_Fe_yp_/Al matrix composite, but lots of small voids still existed [11]. So it is suggested that the tensile elongation, including the tensile strength, can be further enhanced if the voids are decreased or eliminated.

The authors combined the merits of PM and thixoforming and proposed a new technology named powder thixoforming for fabricating the Ti@Al_3_Ti_p_/Al matrix composite [12]. A green powder compact of Ti and Al powders is first obtained through blending and cold pressing of PM, and then is partially remelted to a semisolid state of the Al matrix alloy and thixoformed. The core-shell-structured Ti@Al_3_Ti_p_ can be achieved through reaction between the Ti powder and Al melt during the partial remelting. In addition, liquid phase can fill the voids during the subsequent thixoforming, and thus, a compact-microstructure Ti@Al_3_Ti_p_/Al matrix composite component with near-net shape is achieved. Furthermore, the reaction time needed for forming the core-shell-structured reinforcing particles can be obviously shortened due to the elevated temperature compared with PM technology. That is, powder thixoforming is a promising way to fabricate and form Ti@Al_3_Ti_p_/Al matrix composite components with high performance.

The authors investigated the microstructural evolutions of Ti-A356 and Ti-2024 compacts during partial remelting, and the effects of alloying elements such as Si, Cu, Zn, and Mg on the formation of the Ti@Al_3_Ti_p_ [12,13,14]. The results indicated that Si element participated in the reaction between Ti powders and Al melt, and an ideal core-shell-structured Ti@(Al-Si-Ti)_p_ with a thick and compact Al-Si-Ti compound shell was obtained in A356 alloys after being properly heated at semisolid temperatures. More importantly, the authors studied the effects of one main processing parameter—reheating time at semisolid temperature of 600 °C—on the microstructure and tensile properties of the resulting composites [15]. The results showed that the Ti@(Al-Si-Ti)_p_/A356 matrix composite formed at reheating for 50 min had an ultimate tensile strength (UTS) of 371 MPa, yield strength (YS) of 268 MPa, and elongation of 8.3%. The UTS and YS were equivalent to those of the monolithic (Al,Si)_3_Ti_p_/A356 matrix composite thixoformed when the Ti powders had completely reacted, but the elongation was increased by 167.8%. To our knowledge, this composite has the lowest elongation reduction and the highest UTS increment compared with the other A356 matrix composites reinforced by Al_3_Ti_p_ [5], SiC_p_ [16], TiB_2p_ [17,18,19], Al_2_O_3p_ [20,21], BC_4p_ [22], and Mg_2_Si_p_ [23]. This demonstrates that the powder thixoforming is suitable for preparing and forming PRAMCs with good performance.

For the powder thixoforming technology, reheating temperature, mould temperature, punch velocity, and pressure are equally important processing parameters with the reheating time. So far, only one paper studied the effects of reheating time on the microstructure and tensile properties of the Ti@(Al-Si-Ti)_p_/A356 matrix composite as mentioned above [15]. Therefore, the effects of reheating temperature were investigated in this work. Especially, the influences on the reinforcing particle behaviors during tensile testing, strengthening, and toughening mechanisms were mainly discussed.

## 2. Experimental Procedure

The A356 alloy powder used in the work was composed of pure Al powder and Al-15Si-Mg alloy powder, and the average composition was consistent to that of A356 alloy: 7.51 wt % Si, 0.217 wt % Mg, 0.115 wt % Fe, 0.097 wt % Ti, and balance Al. The average size of the powders was 16.23 μm. The Ti powder had a purity 99.99 wt % and a size of 18.65 μm. To replenish the consumption of Al element from the reaction with Ti, an extra amount of pure Al powder (purity of 99.98 wt % and average diameter of 11.82 μm) was added. These three kinds of powders were all made by atomization.

40 g A356 powder, 3.48 g pure Al powder and 2.05 g Ti powder were put into each ball pot of a ND7-21 planetary ball-milling machine (Nanjing Levinstep Technology Co., Ltd., Nanjing, China) and blended. The Ti amount was according to theoretically generating 10 vol % Al_3_Ti when the Ti powder completely reacted and the pure Al powder amount was according to Al/Ti atom ratio of 3:1. The employed ball-to-powder weight ratio, rotation time, and mixing time were 5:1, 100 rpm, and 40 min, respectively. The powder mixture was then cold-pressed into green compacts with dimensions of Φ 45 mm × 16 mm using an Y32-60T hydraulic machine. Subsequently, these green compacts were promptly put into a SK-G08123K-HD tubular vacuum furnace (Tianjin Zhonghuan Furnace Co., Ltd., Tianjin, China) with different preheating temperatures (i.e., semisolid temperatures of A356 alloy) and heated for 50 min, respectively. The used preheating temperatures were 590 °C, 600 °C, 605 °C, and 610 °C. The temperature control accuracy of the furnace was at ±1 °C. The employed vacuum was less than 10^−2^ Torr. The previous investigation indicated that heating for 50 min at 600 °C could obtain ideal core-shell-structured Ti@(Al-Si-Ti)_p_ and the resulting composite had high performance [15], so the reheating time in this work was set to 50 min. Finally, the heated compacts were quickly handled into a mould with a cavity of Φ 50 mm × 30 mm and thixoformed at a pressure of 150 MPa respectively. The preheating temperature of the mould was 200 °C.

Metallographic specimens were machined from the center regions of the thixoformed products, and then ground, finished, polished, and finally etched by 4 wt % NaOH aqueous solution. They were then observed and analyzed on a MEF-3 optical microscope (OM, Leica Microsystems, Vienna, Austria), QUANTA FEG 450 scanning electron microscope (SEM, FEI, Hillsboro, OR, USA), and energy disperse spectroscopy (EDS, Oxford instruments, Oxford, UK) equiped on this SEM. To verify the phase constituents, they were also analyzed on a D/max-2400 X-ray diffraction analyzer (XRD, Rigaku, Tokyo, Japan). In order to quantitatively examine the primary particle size and fraction of secondarily solidified structures (for the convenience, the phrase of secondarily solidified structures was abbreviated as 3Ss), the SEM images with magnification of 500× were analyzed using Image-Pro Plus 6.0 software (Media Cybernetics Company, Silver Spring, MD, USA). For each specimen, at least three images were measured. The relative densities of the products were measured by Archimedes drainage method to reveal their microstructure compactness. At least five tensile bars with a gauge of 10 mm and a cross-section of 2.5 mm × 1.5 mm were machined from the center region of each product, and tensile testing was carried out on a WDW-100D universal material testing machine (KASON Testing equipment Co., Ltd., Jinan, China) at a loading rate of 8.33 × 10^−3^ mm·s^−1^. Typical fracture surfaces and their side-views were also observed on the SEM and OM, respectively. In situ tensile testing was performed on the SEM at a loading rate of 3 × 10^−4^ mm·s^−1^ in order to further verify the effects of the reheating temperature on the fracture process during tensile testing, and thus, the corresponding strengthening and toughening mechanisms.

## 3. Results and Discussion

### 3.1. Effect on Microstructure

**Figure 1:**
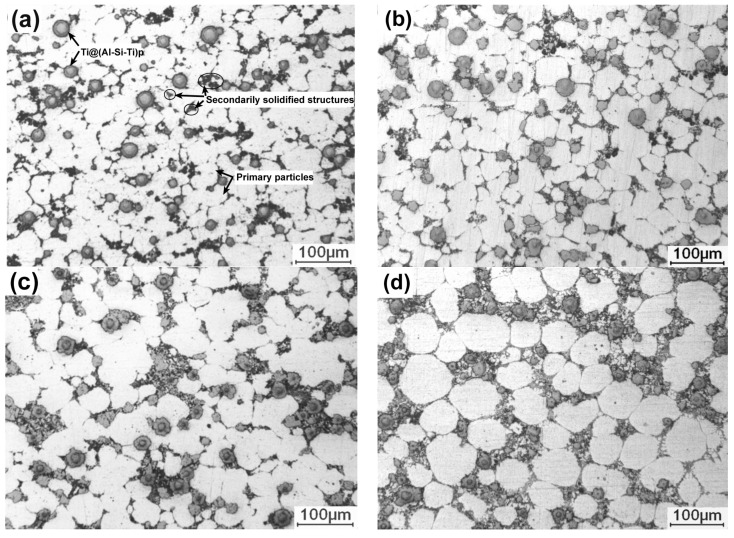
OM images of the composites thixoformed at reheating temperatures of (**a**) 590 °C, (**b**) 600 °C, (**c**) 605 °C, and (**d**) 610 °C.

**Figure 2:**
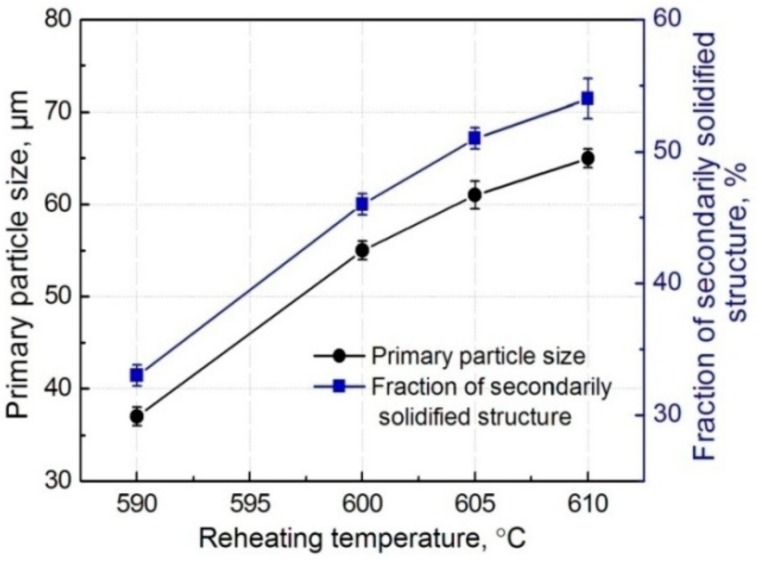
Variations of primary particle size and secondarily solidified structure amount vs reheating temperature.

Figure 1 shows the microstructures of the Ti@(Al-Si-Ti)_p_/A356 matrix composites thixoformed at reheating for 50 min at different temperatures. It can be seen that all of the microstructures are composed of primary particles, 3Ss and Ti@(Al-Si-Ti)_p_. The Ti@(Al-Si-Ti)_p_ distribute in the regions between the primary particles, i.e., in the 3Ss, and their distribution is quite uniform at the reheating temperatures of 590 °C and 600 °C (Figure 1a,b). But they tend to agglomerate as the temperature rises (Figure 1c,d). The primary particles at 590 °C are in a form of interconnected small particles (Figure 1a), and are separated by the 3Ss accompanied with their growth, evolving into the individual spheroidal particles with an average size of about 55 μm at 600 °C (Figure 1b). But they then tend to agglomerate to form large-sized interconnected irregular particles when the temperature is further elevated (Figure 1c), and finally become large spheroidal particles at 610 °C (Figure 1d). In general, the primary particles show a continuous growth trend with rising the temperatures. In addition, the 3Ss amount also steadily increases all the while (comparing the images in Figure 1). As shown by Figure 2, the quantitative examinations more clearly indicate that both the primary particle size and 3Ss fraction all continuously increase as the reheating temperature rises. In general, the composite thixoformed at 600 °C has a typical semisolid forming microstructure with middle-sized primary particles and uniformly-distributed reinforcing particles.

It is known that reheating temperature mainly determines the liquid fraction of a semisolid ingot. The higher the temperature is, the more the liquid phase is, and thus, the larger the amount of 3Ss that solidifies from the liquid phase is. At the temperature of 590 °C, the lowest temperature used in this work, the primary particles cannot be completely separated by liquid phase because there is not enough liquid phase, resulting in the interconnected small-particle structures (Figure 1a). As the temperature rises, the primary particles are gradually separated by the increased liquid phase, i.e., the primary particles are separated by the 3Ss (Figure 1b). During partial remelting, the size decrease of primary particles from their partial melting and the size increase from mergence and/or Ostwald ripening are in a dynamic competition status [13,14]. The present results indicate that the former factor is smaller than the latter one, and thus, the primary particle size continuously increases. The mergence is determined by the coupled effect of solid fraction and temperature, high solid fraction promotes the mergence, but low temperature at this condition slows the mergence due to the low atom diffusion rate. Figure 1c implies that the mergence is quite active at 605 °C, resulting in the formation of the large irregular primary particles. But when the temperature exceeds 605 °C, Ostwald ripening becomes the main coarsening mechanism in view of their individual and spheroidal morphology (Figure 1d). In addition, the increase of liquid phase and the coarsening of primary particles must give rise to the micro-segregation of liquid or solid phase, and thus, results in the aggregation of reinforcing particles that distribute in the liquid phase.

**Figure 3:**
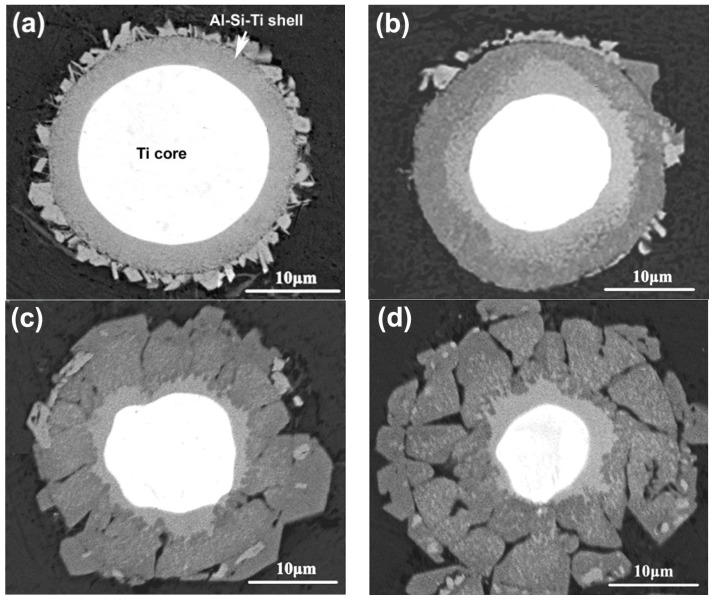
SEM images of reinforcing particles in the composites thixoformed at reheating temperatures of (**a**) 590 °C, (**b**) 600 °C, (**c**) 605 °C, and (**d**) 610 °C.

To verify the evolution process of the reinforcing particles with the reheating temperature, the specimens were also observed by SEM, as shown in Figure 3. It is seen that the reinforcing particle at 590 °C presents a well core-shell-structure with a uniform and compact light grey shell in addition to a round of jagged structures (Figure 2a). As the temperature rises, the reaction shell thickens and the outside jagged structures coarsen (Figure 3b–d). Additionally, a kind of grey structure is formed from the outside of the light grey shell and the shell evolves into a dual-layer structure at 600 °C (Figure 2b). Subsequently, some radial cracks generate within the thickened shell and the reinforcing particle becomes a structure that is composed of a small-sized core-shell-structured core (with a thin light grey shell) and surrounding cracked grey shell at 605 °C (Figure 3c). Finally, the cracked grey structures peel off and the center part evolves into a smaller core-shell-structured particle, which is similar to the reinforcing particle at 605 °C, when the temperature rises to 610 °C (Figure 3d). So it can be concluded that rising reheating temperature thickens the shells, but also promotes the shells to fracture. Reheating at 590–600 °C for 50 min can obtain perfect-morphology core-shell-structured reinforcing particles. In addition, elevating the temperature possibly facilitates the first formed compound shell to transform into another phase.

**Figure 4:**
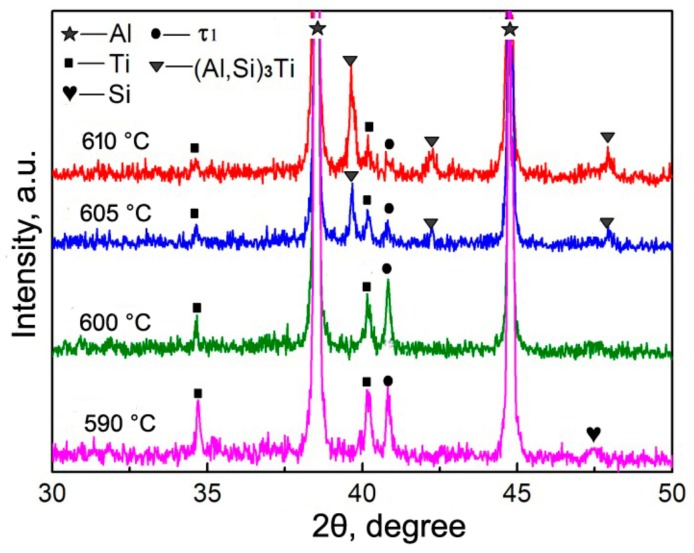
XRD patterns of the composites thixoformed at different reheating temperatures.

The white cores in Figure 3 should be the residual Ti and the surrounding shells or other-morphological structures, including the outside jagged structures, are reaction compounds between the Ti powders and Al melt. The jagged structures originated from the reaction of TiO_2_ film on the Ti powder surface with Al melt, and they belong to Al_2_O_3_-containing Al-Si-Ti compound [12]. The XRD results indicate that only one compound phase of τ1 (Al_5_Si_12_Ti_7_) has formed at 590 °C, besides Al, Ti, and Si phases in the original powders (Figure 4), which implies that the light grey shell in Figure 3a is τ1 phase and the out jagged structures belong to Al_2_O_3_-containing τ1 phase. When the temperature rises, another Al-Si-Ti ternary phase of (Al,Si)_3_Ti generates besides τ1 phase, and its diffraction intensities become more and more intensive, while those of τ1 phase get weaker and weaker (Figure 4). It is found that the diffraction peaks of (Al,Si)_3_Ti andτ1 phases are all not clear at 600 °C. This should be contributed to their relatively low concentrations. So it can be deduced that the light grey layers close to the Ti cores in Figure 3b–d are τ1 phase and the out grey layers or cracked shells belong to (Al,Si)_3_Ti phase. That is, the first formed τ1 phase gradually transforms into (Al,Si)_3_Ti phase from the shell outside to inside as the reheating temperature rises.

**Figure 5:**
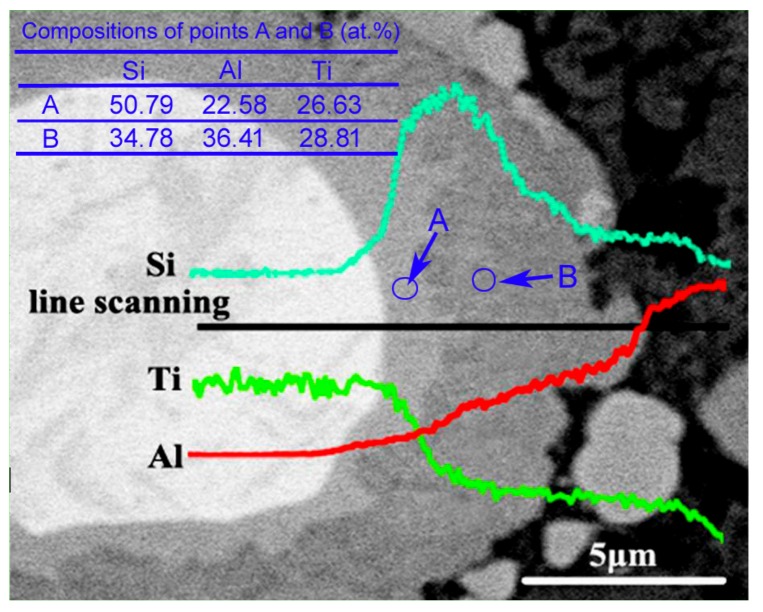
EDS analysis of a reinforcing particle formed at reheating temperature of 600 °C.

The EDS result of the reinforcing particles at 600 °C indicates that the inner light grey layer is rich in Si, while the out grey layer is rich in Al, and the Al concentration gradually increases towards the shell outside, while the variation of Si content is just opposite (Figure 5). The quantitative examination shows that the Si concentration in the light grey layer (point A) is obviously higher than that in the grey layer (point B). This is consistent to the result that τ1 phase has higher Si content than (Al,Si)_3_Ti phase [24]. (Al,Si)_3_Ti phase is formed from partial substitution of Al atoms in Al_3_Ti phase by Si atoms and its composition varies in a wide range, but the crystal still maintains the tetragonal structure of Al_3_Ti phase [25]. In fact, the composition of τ1 phase also varies widely, although its accepted chemical formula is expressed as Al_5_Si_12_Ti_7_ [24]. When the Si content in (Al,Si)_3_Ti phase exceeds 15.07%, it will transform into τ1 phase [24,25]. As discussed above, rising the reheating temperature not only generates more reaction product, and thus, consumes more Si element, but also generates more liquid phase from partial melting of primary Al particles. These two results must lead the concentration of Si in the Al melt to decrease, and then part of Si atoms in τ1 phase diffuses into Al melt, resulting in the transformation of τ1 phase with high Si content into (Al,Si)_3_Ti phase with low Si content. This is demonstrated by the XRD results shown in Figure 4, Si phase cannot be detected when the temperature exceeds 600 °C. In addition, this transformation should start from the interface of τ1 phase/Al melt and develop towards the shell inside. In view of thermodynamics, (Al,Si)_3_Ti phase is more stable than τ1 phase at elevated temperatures [26]. So rising the temperature facilitates τ1 phase to transform into (Al,Si)_3_Ti phase. Furthermore, it can be seen that the newly formed product with Ti is always τ1 phase from Figure 4 and Figure 5, regardless of the reheating temperature. The reason is that the Si atom has a greater affinity with Ti than with Al [24]. So we can conclude that the newly generated product of Ti with Al melt is always τ1 phase, but it will transform into (Al,Si)_3_Ti phase due to the decreased Si content in Al melt and its increased thermodynamic instability with rising the reheating temperature.

It is known that volume expansion occurs during transformation of Ti into Al-Ti compounds [27]. It is due to the reason that stress concentration generates in the shells and increases with the shell thickening. The shells thereby crack when the stress exceeds their strength and then peel off. Our previous investigation on the 2024Al-Ti system proposed that stress reached 15.89 GPa when a 2 μm Al_3_Ti shell formed around a 9.28 μm Ti powder, which was higher than the theoretical strength of Al_3_Ti phase (14.4 GPa) [13]. In addition, the difference in volume expansion ratio was very large for forming different compounds, i.e., 2.55 for Al_3_Ti, 1.8 for τ1 phases (according to the composition of Al_5_Si_12_Ti_7_) and a value varied between 2.55 and 1.8 for (Al,Si)_3_Ti with its composition [14]. That is, the generated stress in (Al,Si)_3_Ti phase is larger than that in τ1 phase. As discussed above, both the shell thickness and the proportion of (Al,Si)_3_Ti phase increase as the reheating temperature rises. So the stress concentration increases by elevating the reheating temperature and the shells will crack as the temperature rises to a given value.

In summary, both the primary particle size and 3Ss amount in the composite increase by rising the reheating temperatures, accompanied by the agglomeration of reinforcing particles. Simultaneously, the reaction shells thicken, but fracture and peel off from the Ti cores when the temperature exceeds 600 °C. In addition, the firstly formed τ1-phase shells gradually transform into (Al,Si)_3_Ti phase from the outside to the inside. At 600 °C, a typical semisolid-forming microstructure with middle-sized primary particles and uniformly-distributed spheroidal core-shell-structured reinforcing particles with a thick, uniform, and compact dual-phase Al-Si-Ti compound shell are achieved.

### 3.2. Effects on Tensile Properties

**Figure 6:**
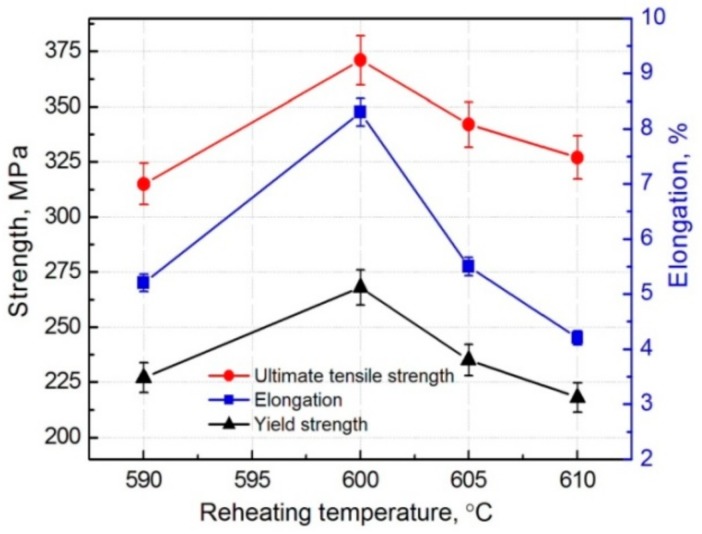
Variations of tensile properties of the composite vs reheating temperature.

Figure 6 presents the variations of tensile properties of the composite with the reheating temperature. It shows that the ultimate tensile strength (UTS), yield strength (YS), and elongation all first increase as the temperature rises from 590 °C to 600 °C, and then continuously decrease. The UTS, YS, and elongation all reach the peak values of 371 MPa, 268 MPa, and 8.3% at 600 °C, respectively.

**Figure 7:**
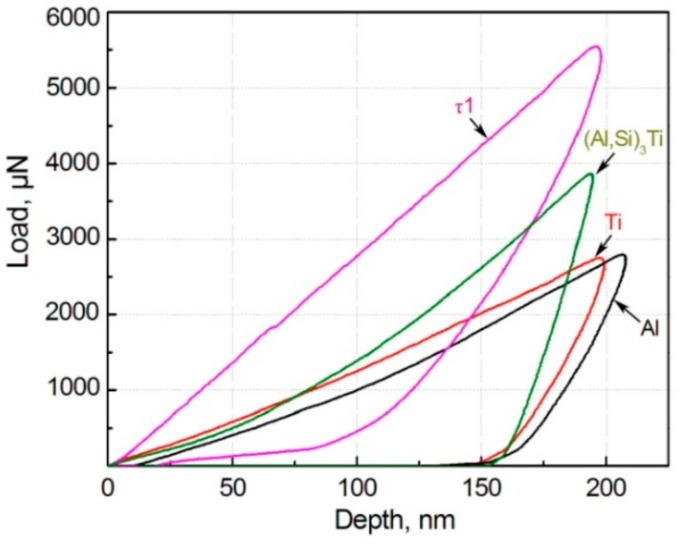
Plot of load vs depth of different phases in the reinforcing particles and Al matrix evaluated by a nanoindenter.

According to the microstructure characteristics discussed in the above section, the factors for affecting the tensile properties mainly include three aspects, the primary particle size, 3Ss amount and strengthening role of the reinforcing particles. First, the primary particle size continuously increases as the reheating temperature rises, which must decrease the tensile properties. Second, the 3Ss amount also increases with rising the temperature, which indirectly implies that the liquid fraction increases in the semisolid ingot prior to thixoforming. So the mould-filling ability and feeding capacity to solidification shrinkage during thixoforming, and thus, the microstructure compactness of the resulting composite is improved. Table 1 gives the relative densities of the composites thixoformed at different reheating temperatures. It indicates that the density increases when the temperature rises from 590 °C to 600 °C. However, the feeding capacity is impaired due to the formation of the irregular large-sized interconnected primary particles as the temperature further rises (Figure 1c), resulting in the decrease of the density (Table 1). In addition, the solidification behavior becomes closer to that of complete liquid melt due to more liquid formation. So the possibility for forming porosity is enhanced, also leading the density to decrease. Third, the total thickness of the shells increases and the first-formed τ1-phase shells gradually transforms into (Al,Si)_3_Ti phase as the temperature rises. The (Al,Si)_3_Ti phase is softer than the τ1 phase as shown by Figure 7, but it is significantly harder than the Ti core and Al matrix. Table 2 gives the values of the microhardness and elasticity modulus of the different phases in the composite. So the increase in shell thickness with raising the temperature from 590 °C to 600 °C must enhance the strengthening role of the reinforcing particles. But the strengthening effect is then impaired when the temperature exceeds 600 °C due to cracking of the shells (Figure 3c). The present result indicates that the enhanced strengthening role from the increased shell thickness and improved microstructure compactness is larger than the weakening effect from the coarsened primary particles when the temperature rises from 590 °C to 600 °C. But all of the three factors have a negative effect on the strengthening role when the temperature exceeds 600 °C.

**Figure 8:**
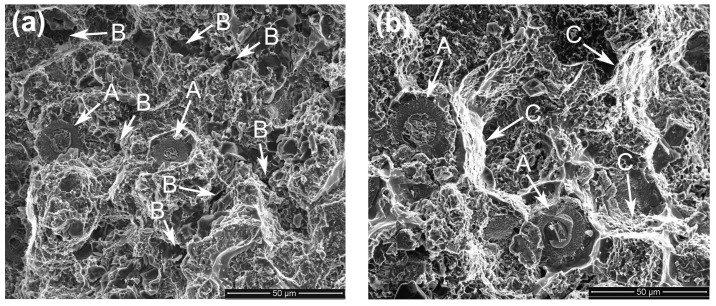

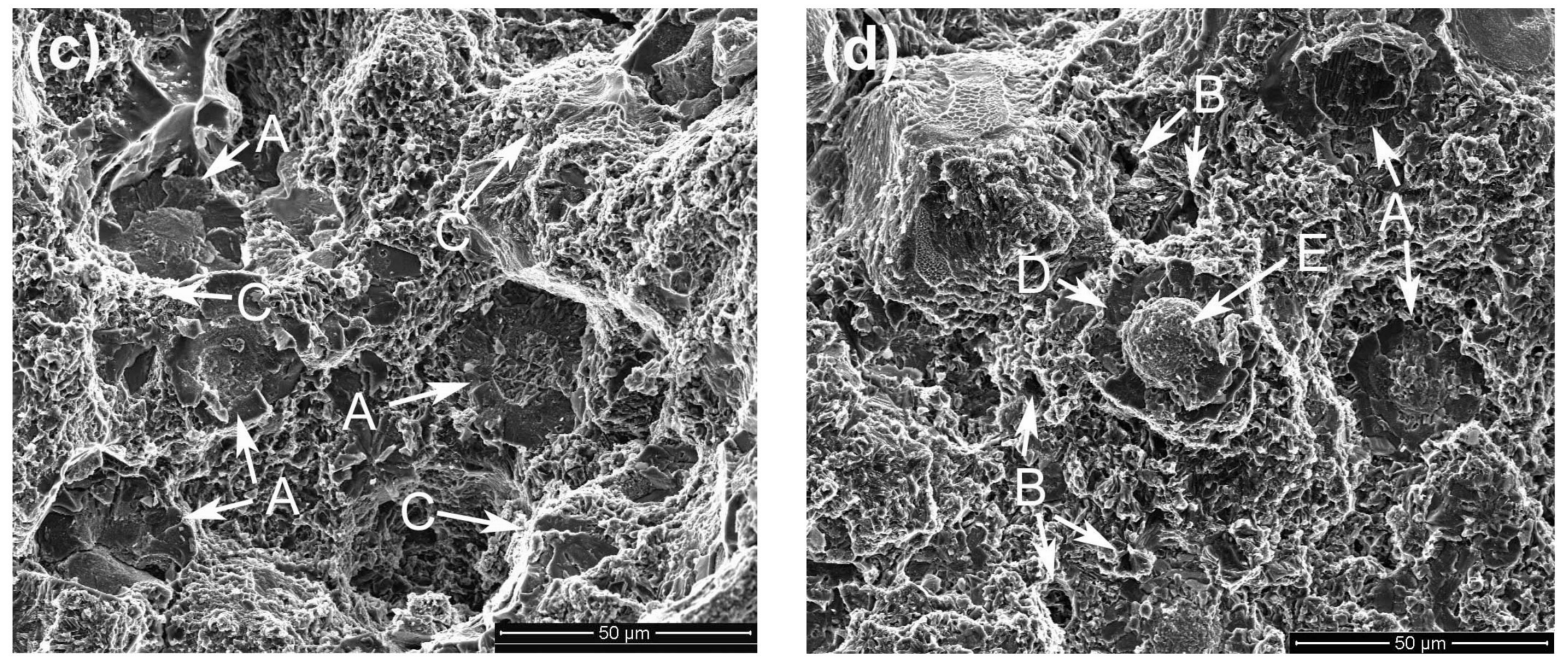
Fractographs of the composites thixoformed at reheating temperatures of (**a**) 590 °C, (**b**) 600 °C, (**c**) 605 °C, and (**d**) 610 °C.

Figure 8 presents the typical fractographs of the composites. It shows that there is no obvious difference between them and they are all composed of torn matrix and fractured reinforcing particles (marked by arrows A). But three small differences are found through careful comparison. First, there are lots of small pores on the fracture surfaces of the composites thixoformed at 590 °C and 610 °C (marked by arrows B in Figure 8a,d). This further demonstrates the variation of the microstructure compactness with the reheating temperature discussed above, and is consistent to the density variation (Table 1). Second, the tearing trace of the matrix is more obvious for the composites thixoformed at 600 °C and 605 °C (marked by arrows C in Figure 8b,c). This implies that these two composites should have high tensile properties, especially high elongation (Figure 6). The reason should be mainly attributed to the improved matrix microstructure compactness. Third, the reinforcing particles for the composites formed at 590 °C–605 °C frequently fracture across their center region (marked by arrows A in Figure 8a–c). But for the composite formed at 610 °C, some of them fracture in a dual-form, the surrounding shells fracture across themselves (marked by D in Figure 8d), while the center cores debond from the shells (marked by E).

**Figure 9:**
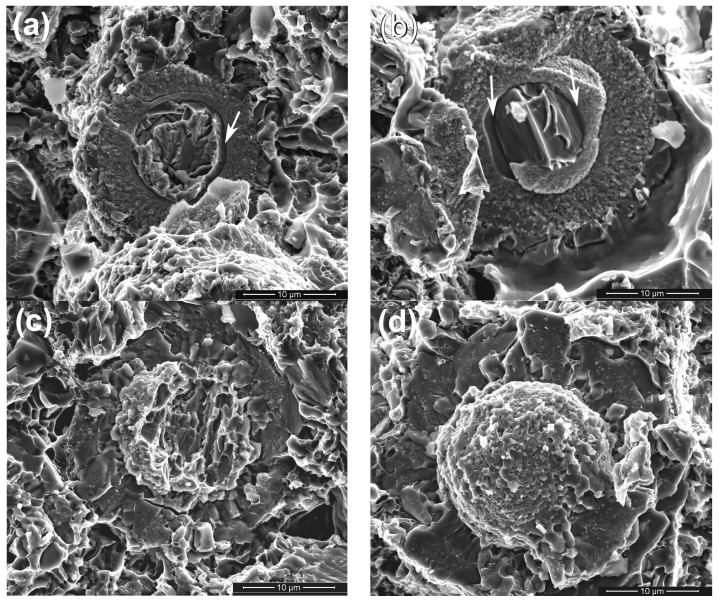
Morphologies of reinforcing particles on the fracture surfaces of the composites thixoformed at reheating temperatures of (**a**) 590 °C, (**b**) 600 °C, (**c**) 605 °C, and (**d**) 610 °C.

The typical morphologies of the visible reinforcing particles on fracture surfaces are more clearly shown in Figure 9. It is obviously seen that the reinforcing particles for the composites thixoformed at 590 °C and 600 °C fracture through their center cores (Figure 9a–c), while some of them for the composite at 610 °C only fracture across the outside shells and the cores debond from the shells to form a bulge (Figure 9d). Figure 9a and b also show that the shells at 590 °C and 600 °C are quite compact and the later one is obviously thicker than the former one. But the shells at 605 °C and 610 °C have cracks or have blocky characteristics (Figure 9c,d). These microstructural features are completely consistent with the results from the metallographic observation in Section 3.1. While the shells for the composite formed at 605 °C fracture, the bonding strength with the Ti cores, similar to those for the composites formed at 590 °C and 600 °C, should be quite strong, and thus, the reinforcements also frequently fracture across the center Ti cores. But the strengthening role of such reinforcements is smaller than that of the reinforcements with equivalent, even thinner, compact shells. As shown by Figure 3d, the reinforcement at 610 °C is composed of a core-shell-structured particle core and surrounding compound particles. The bonding strength between these two parts should be relatively low, and thus, debonding is easy to occur and their strengthening role should be smaller than that of the ones with crack-containing shells. In addition, obvious gaps or cracks can be seen between the Ti core and compound shell for the composites formed at 590 °C and 600 °C (marked by arrows in Figure 9a,b). This implies that the interface bonding between them is not so strong that partial debonding can occur during tensile testing. But the complete debonding, like that shown by Figure 9d, is not found. All of these further demonstrate that the strengthening role of the reinforcements increases as the temperature rises, but decreases when the temperature exceeds 600 °C.

**Figure 10:**
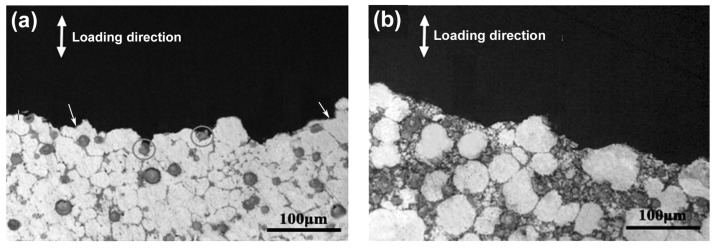
Side-views of fracture surfaces of the composites thixoformed at reheating temperatures of (**a**) 590 °C and (**b**) 610 °C.

The observation for the side-views of the fracture surfaces indicates the crack propagation paths of all the composites during tensile testing are basically along the 3Ss between the primary particles, as shown by Figure 10. The only difference is that cracks occasionally propagate across some primary particles for the composites thixoformed at 590 °C (marked by arrows in Figure 10a). But this case becomes less and less as the temperature rises, and cracks almost completely propagate along the 3Ss when the temperature reaches 610 °C (Figure 10b). As discussed above, pores such as shrinkage porosities and gas pores all distribute in the 3Ss. So the 3Ss are the weak points of the composites, and thus, cracks preferentially propagate along these structures. In addition, the primary particles at 590 °C are basically connected to each other (Figure 1a) and they are gradually separated by the 3Ss, and finally evolve into the individual large-sized spheroidal particles at 610 °C (Figure 1d). So it can be expected that cracks can occasionally propagate across some interconnected primary particles for the composites formed at low reheating temperatures, and this situation will decrease due to the separation of the primary particles by 3Ss and completely disappear as the temperature rises to a given degree.

**Figure 11:**
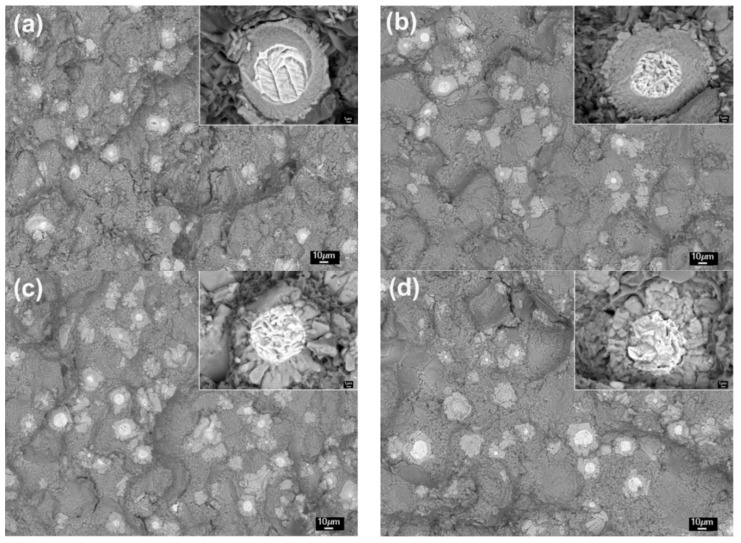
BSE images of fracture surfaces of the composites thixoformed at reheating temperatures of (**a**) 590 °C, (**b**) 600 °C, (**c**) 605 °C, and (**d**) 610 °C. Inserts are the large views of typical fractured reinforcing particles.

**Figure 12:**
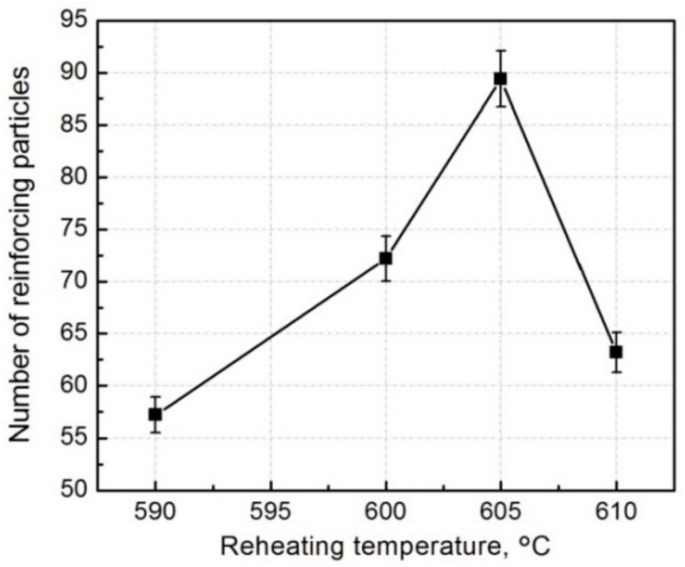
Variation of visible reinforcement number on the fracture surfaces vs reheating temperature.

From Section 3.1 we know that all of the reinforcing particles distribute in the 3Ss for all of the four composites, so it is expected that some reinforcements will fracture when cracks encounter them during propagation, resulting in the visible cracked reinforcements on the fracture surfaces (Figure 8 and Figure 9). The number of the visible reinforcements can thereby reflect the crack propagation path within the 3Ss. For this purpose, the fracture surfaces were also observed by back-scattered electron (BSE) imaging technology, as shown by Figure 11, which intuitively indicates that the number of the visible reinforcements increases as the temperature rises from 590 °C to 605 °C and then decreases when the temperature further rises. The quantitative examination more clearly shows this change tendency (Figure 12). According to the crack propagation discussed above, it can be expected that the number of reinforcements that cracks encounter during their propagation increases with elevating the temperature. But when the temperature rises to a given value, the number then decreases due to the increased 3Ss amount, although cracks still develop along the 3Ss. Based on this analysis, the results from Figure 11 and Figure 12 can be well understood. This further confirms the variation of crack propagation path with the reheating temperature resulted from Figure 10. In addition, the microstructures, especially the shell’s microstructure of the reinforcements can be more clearly observed from the inserts in Figure 11, the shells in the composites thixoformed at 590 and 600 °C are quite compact and the shell in the former composite is thinner than that in the later one, but those in the composites formed at 605 °C and 610 °C have cracks, or are in particle-agglomerates, due to cracking and peeling off.

In general, the tensile properties increase as the reheating temperature rises from 590 °C to 600 °C due to the enhanced strengthening role from the thickened compact shells and densified matrix microstructure, and then decrease when the temperature exceeds 600 °C because of the impaired strengthening effect from the fracture and peeling of the shells, loosened matrix microstructure and coarsened primary particles. The composite formed at 600 °C has peak properties, UTS of 371 MPa, YS of 268 MPa, and an elongation of 8.3%. The visible reinforcing particles on the fracture surfaces always fracture across their center cores, but for the composite formed at 610 °C, some of them fracture only across the outside shells and the cores debond from the outside shells. The failure modes of the reinforcing particles are determined by their microstructures. The number of the visible reinforcements increases as the temperature rises from 595 °C to 605 °C, but then decreases. This change is depended on the variation of matrix microstructure dominated by 3Ss amount and the feature of crack propagation along the 3Ss.

### 3.3. Fracture Process and Toughening Mechanism

**Figure 13:**
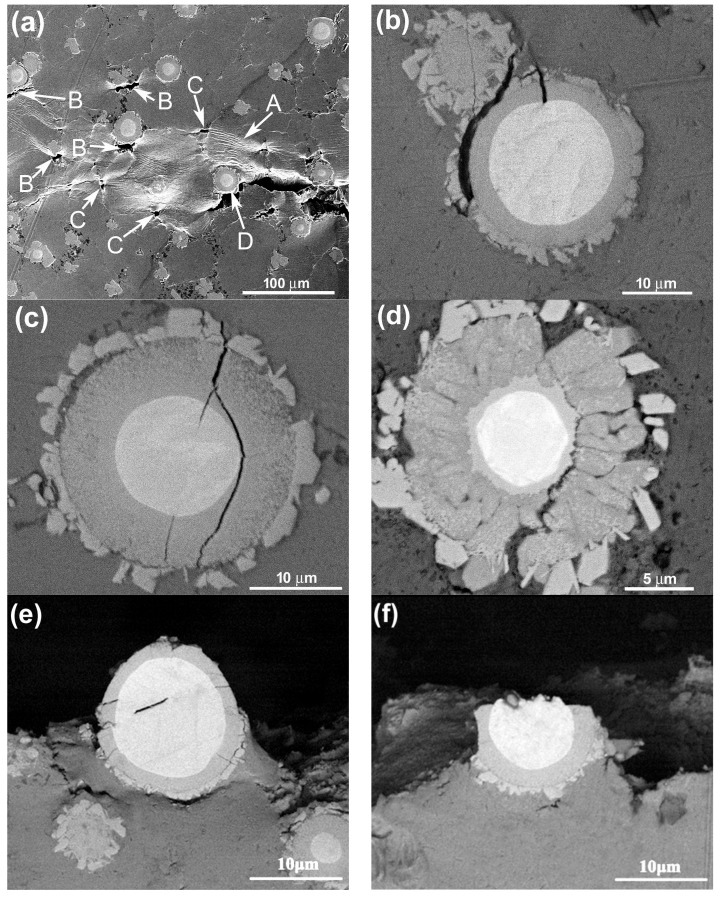
SEM micrographs of the composites thixoformed at reheating temperatures of (**a**–**c**,**f**) 600 °C, (**d**) 605 °C, and (**e**) 590 °C during or after in situ tensile testing.

Figure 13 presents the typical images from in situ tensile testing. It shows that severe plastic deformation occurs in the matrix ahead of the primary crack, resulting in the obvious slip bands (marked by arrow A in Figure 13a). It is just due to the plastic deformation that large stress concentration generates at the reinforcement/matrix interfaces, and small voids then form when the stress is up to a given value (marked by arrows B). As discussed in Section 3.2, the 3Ss are the weak points of the composite, and thus, small voids can also generate in these sites (marked by arrows C). When cracks encounter reinforcement, it will change its propagation direction and bypass the reinforcement (marked by arrow D). So it is suggested that cracks can sometimes bypass the encountered reinforcements, besides frequently propagating across them.

In addition, three kinds of fractured modes of the reinforcements with compact shells are observed in the matrix close to the primary crack. First is that cracks generate between the outer jagged structures and the inner compact shell (Figure 13b). This implies that the bonding strength between these two structures is relatively weak, and thus debonding can occur between them, as shown by Figure 13e. Second is that cracks partially develop around the center Ti core (Figure 13c). This is consistent to the phenomenon shown by Figure 9a,b, partial debonding occurs between the compound shell and Ti core. Third is that the reinforcement crack between the center core-shell-structured core and the outer cracked shell (Figure 13d), which is just matched with the result shown by Figure 8a (marked by arrows D and E) and Figure 9d. However, in most cases, the reinforcements fracture across Ti core, as indicated by Figure 13f. This is corresponded to the situations shown by Figure 9a–c.

**Figure 14:**
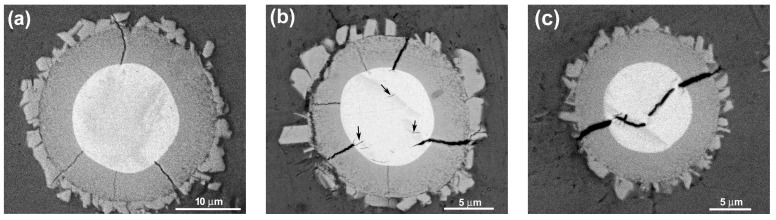
SEM micrographs of crack-having core-shell-structured reinforcing particles in the composites thixoformed at reheating temperatures of (**a**) 600 °C, (**b**) and (**c**) 590 °C after in situ tensile testing.

Figure 14 also shows some typical morphologies of reinforcements in the composites formed at 590 °C and 600 °C after in situ tensile testing. The reinforcement in Figure 14a has only four cracks that radially distribute within the compact shell. However, in Figure 14b, some small cracks appear in the Ti core while the opening gaps of the cracks within the shell are increased. Figure 14c shows that the sizes of the cracks in Ti core also increase in addition to the further enlargement of the opening gaps of the cracks within the shell, and these two kinds of cracks tend to connect with each other in a zigzag way, resulting in the fracture of the reinforcement across Ti core. According to these changes, it is suggested that the sequence of a, b, and c in Figure 14 just show the fracture process of a core-shell-structured reinforcement.

Based on the above discussion, the fracture process of the core-shell-structured particle reinforced Al matrix composites can be deduced. Plastic deformation first occurs in the matrix when the stress is up to the yield strength of the composite (marked by arrow A Figure 13a), resulting in large stress concentration at the reinforcement/matrix interface. Voids then generate at the interface (marked by arrows B) or in the 3Ss (marked by arrows C). Simultaneously, the shells of some reinforcements fracture, forming the radially distributed cracks (Figure 14a). Subsequently, both the voids and cracks gradually grow up. The voids evolve into cracks and develop along the 3Ss. And the opening gaps of the cracks in shells increase, and severe plastic deformation occurs in the Ti cores and Al matrixes. Lots of small cracks then generate in Ti cores due to working hardening (Figure 14b) and some of them grow up and finally connect with the cracks in the shells in a zigzag way (Figure 14c), resulting in the complete fracture of the reinforcements across Ti cores (Figure 9a–c). Occasionally, cracks in shells do not develop within Ti cores, but bypass the Ti cores along their boundaries, as shown by Figure 13c. But for the composites reinforced by the reinforcements with cracked shells (i.e., the composites thixoformed at above 600 °C), cracks do not preferentially develop across Ti cores, but propagate between cracked shells and residual cores (Figure 9d and Figure 13d). The cracks originated in reinforcements then develop towards the Al matrix after the reinforcements completely fracture. When the cracks originated in either the reinforcements or the matrix grows up to a given degree, they will lose stability and rapidly develop [28] and finally connect with each other, resulting in the fracture of the composite. During the growth and subsequent propagation, cracks sometimes bypass the encountered reinforcements in terms of debonding between the outer jagged structures and the inner compact shells (Figure 13b,e).

That is, the first formed cracks in the reinforcements with compact shell are only constrained within the shells. Their sizes are obviously smaller than those generated in monolithic reinforcing particles with the same size to the core-shell-structured ones. So it needs a long process for their sizes to grow up to those of the latter ones due to the hindering effect of the soft Ti cores on their propagation, and thus, quite a large strain should occur for the composite during this process. In addition, the commonly used reinforcements such as SiC, Al_2_O_3_, TiC, TiB_2_, and Al_3_Ti are always in an irregular morphology with sharp edges, and large stress concentration generates early at these edges and leads the reinforcements to prematurely fracture [3,18,21]. But for the core-shell-structured reinforcing particles in the present work, their morphology is very spheroidal (Figure 3a,b and Figure 14), and thus, stress concentration generated at the interfaces with matrix is relatively small, and thus, their fracture is delayed. Therefore, the deferred formation of cracks and their small size are the main reasons for the ductility improvement of the Al matrix composite reinforced with core-shell-structured reinforcing particles.

In general, small voids always first generate at the interfaces of core-shell-structured particle/Al matrix and in 3Ss during tensile testing. Simultaneously, the shells of some reinforcements also fracture and small cracks generate in the shells in a radial distribution. The small voids then grow into cracks and develop along the matrix, while the opening gaps of the cracks in the shells increase and the Ti cores experience a process of significant plastic deformation, formation and subsequent growth of small cracks, and finally the grown cracks connect with the cracks in the shells in a zigzag way, resulting in the fracture of the reinforcements across the Ti cores. Occasionally, cracks in the shells also develop around the Ti cores. For the composites thixoformed at high reheating temperatures, cracks also propagate between the outer cracked shell and inner core. As the cracks originated from both the matrix and reinforcements grow to a given degree, they then lose stability and rapidly propagate, leading to the fracture of the composite. Cracks sometimes bypass the reinforcing particles along the regions between the outer jagged structures and the inner compact shell. The delayed formation of cracks in the reinforcing particles and their small size are contributed to the ductility improvement of the composite.

### 3.4. Strengthening Model

At present, all of the existing strengthening models for PRAMCs are aimed at the composites reinforced by monolithic single-phase particles and the most acceptable one to predict YS, σ_cy_, is the modified shear lag model (MSL), which is based on the load transfer mechanism, and can be expressed as [29]:

(1) $$\sigma_{\mathrm{cy}}=\left(\sigma_{0}+\Delta \sigma_{\mathrm{GR}}+\Delta \sigma_{\mathrm{GND}}+\Delta \sigma_{\mathrm{TMS}}+\Delta \sigma_{\mathrm{SS}}+\sigma_{\mathrm{OS}}\right)\times \left(\frac{V_{p}\left(S+2\right)}{2}+1-V_{p}\right)$$

where σ_0_ is the YS of unreinforced matrix alloy; V_p_ and S is the volume fraction and aspect ratio of reinforcements respectively; Δσ_GR_, Δσ_GND_, Δσ_TMS_, Δσ_SS_, and Δσ_OR_ are the strength increments resulted from grain refinement (GR), geometrically necessary dislocations (GND), thermal mismatch strain (TMS), solid solution (SS), and Orowan strengthening (OS), respectively, due to the introduction of reinforcements, and are presented as [29,30]:

(2) ΔσGR=K(d)12

(3) $$\Delta \sigma_{\mathrm{GND}}=\frac{2G(1-{\upsilon )\varepsilon V}_{p}}{\left(1-2\upsilon \right)}$$

(4) ΔσTMS=αGb(12ΔTΔCVpbdr(1−Vp))12

(5) ΔσSS=Gε’(Δxf4)12

(6) ΔσOS=2Gb0.6d(2πVp)12

where K is the material coefficient and d is the grain size; G is the shear modulus of matrix, ε is the yield strain of composite, and υ is the Poisson ratio of matrix; α is a constant, b is the Burgers vector, ΔT is the temperature interval from room temperature to the processing temperature, d_r_ is the average diameter of reinforcements, and ΔC is the difference of coefficient of thermal expansion (CET) between matrix and reinforcement; ε’ is the fractional difference in atomic diameter between solute atoms and matrix atoms, and Δx_f_ is the solute concentration difference between grains in composite and matrix alloy.

As for the multi-phase or multi-structure particle reinforced metal matrix composites, i.e., the core-shell-structured particle reinforced composites, there is only one paper from the authors, which predicted the YS of the Ti@(Al-Si-Ti)_p_/A356 matrix composites thixoformed under different reheating durations at 600 °C, based on the above model [15]. As discussed in Section 3.2, the strengthening role of this kind of reinforcements varies with the reheating temperature because their microstructure and phase constituent change with the temperature. To make this model suitable to this kind of composites, the authors introduced a coefficient of C to modify the volume fraction of reinforcement V_p_ and substituted it using the equivalent volume fraction $V_{p}^{*}$:

(7) $$V_{p}^{*}={\mathrm{CV}}_{p}$$

(8) $$V_{p}=n\left[V_{2}+f\left(V_{1}-V_{2}\right)\right]$$

(9) $$C=\frac{A_{1}E_{1}{+A}_{2}E_{2}}{E_{1}}$$

where n is the number of original Ti particles per unit volume, f is the expansion factor during Ti transformation into Al-Si-Ti compounds (using the value of transforming into Al_3_Ti), V_1_ and V_2_ are the volumes of one original Ti particle and residual Ti core in a Ti@(Al-Si-Ti)_p_, respectively; A_1_ and A_2_ are the volume fractions of Al-Si-Ti compounds and Ti core in a Ti@(Al-Si-Ti)_p_, respectively, E_1_ and E_2_ are the elasticity moduli of Al-Si-Ti compounds (using the value of Al_3_Ti) and Ti, respectively. The role of OR can be neglected because it has effect only when the reinforcement size is less than 1 μm [30]. According to Formulas (7) and (9), it can be found that $V_{p}^{*}$ increases with the increase of shell thickness (i.e., the increase of A_1_), and thus, the strengthening role to the matrix is improved. This is consistent with the experimental results from both the present work and Reference [15], the thicker the compact shells are, the higher the YS will be.

To further verify the rationality of this MSL model modified according to the characteristics of core-shell-structured particle reinforced metal matrix composites, the theoretical calculations were also carried out in this work. In addition, the contributions of each strengthening mechanism subjected to the reheating temperatures were discussed.

**Figure 15:**
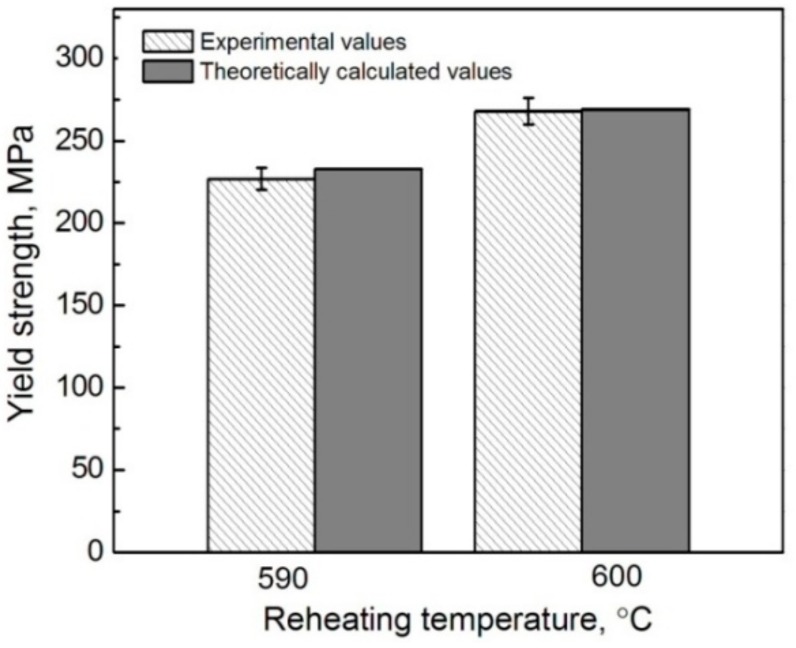
Experimental and theoretically calculated YS values of the Ti@(Al-Si-Ti)_p_/A356 composites thixoformed at reheating temperatures of 590 °C and 600 °C.

Substituting V_p_ in Formulas (1), (3), and (4) by $V_{p}^{*}$, expressed by Formula (7), then incorporating Formulas (2)–(5) into Formula (1), and finally taking the data in Table 3 and Table 4 into Formula (1), the YS values of the composites thixoformed at different reheating durations can be obtained. It is noted that only the shells in the composites formed at 590 °C and 600 °C are compact, so the YS values of these two composites were calculated. As shown by Figure 15, the calculated values are well consistent to the experimental ones, which further confirms that the modified MSL model is actually suitable to predict the YS of this kind of composite, and especially, the considered strengthening mechanisms such as GR, GND, TMS, and SS are also reasonable.

**Figure 16:**
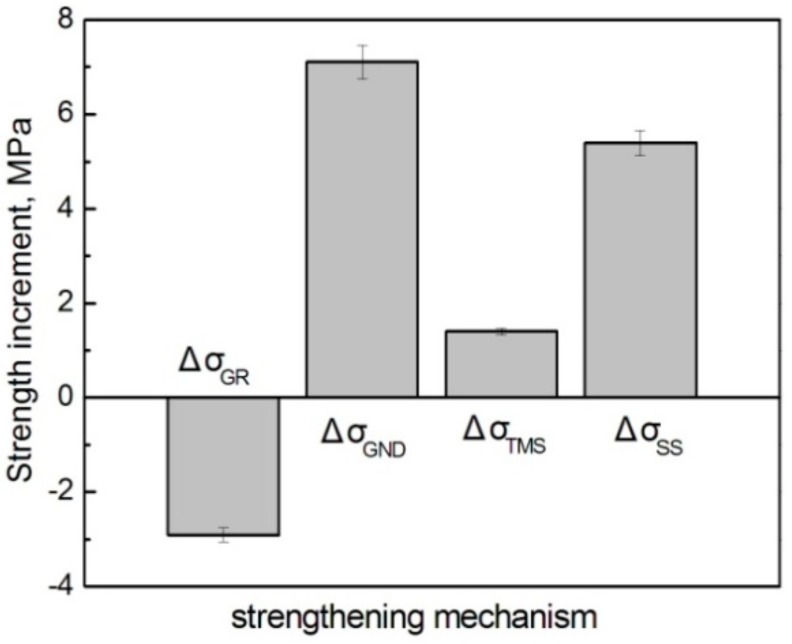
Strength increments from different strengthening mechanisms when the reheating temperature rises from 590 °C to 600 °C.

Table 5 gives the respective contributions of GR, GND, TMS, and SS to the strength increment of the composites formed at 590 °C and 600 °C. It is found that the largest contribution is not from the expected reinforcements themselves, but from the SS strengthening of the Ti element, and this contribution is further enhanced as the reheating temperature rises. This implies that the SS strengthening of the Ti element to Al alloy is quite large, although its solid solubility is very small (0.272–0.393 at % at 590–600 °C, as shown in Table 4 and Al-Ti binary phase diagram [37]). The contribution increase is attributed to the solubility increase of Ti from 0.272 at % to 0.393 at % as the temperature rises from 590 °C to 600 °C. The strengthening from GR is in the second place at 590 °C, but drops to the third place at 600 °C due to the coarsening of primary particles from 37.4 μm to 44.2 μm. However, the effect of GND is sharply increased and rises to second place because of the improved strengthening role of the reinforcements originating from the thickened shells. As implied by the correction factor of C (Formula (9)), the elastic modulus or stiffness of the reinforcements should increase as their shell thickness increases from 4.8 μm to 8.45 μm (calculated according to (d_r_-d_Ti_)/2 in Table 4) with rising the reheating temperature from 590 °C to 600 °C, so the plastic shear strain gradient, and thus, the resulting dislocation density in the matrix surrounding the reinforcements should be enhanced [38], resulting in the significant increase of Δσ_GND_ from 9.9 MPa to 17 MPa. In addition, the dislocation density induced by CTE mismatch is also increased due to the enhanced elasticity modulus [34], so the contribution from the TMS strengthening is also increased. Figure 16 presents the strength increments from the different strengthening mechanisms, which clearly shows that the contribution from GND is the largest, followed by those from SS and TMS, while the contribution from GR is negative.

Therefore, it can be concluded that the modified MSL model that incorporates with the indirect strengthening mechanisms such as GR, GND, TMS, and SS, and introduces a correction factor of C that is actually suitable for accurately predicting the YS of the core-shell-structured Ti@(Al-Si-Ti)_p_ reinforced Al matrix composites. Unexpectedly, the largest contribution to the YS is not the result of the strengthening mechanisms related to the formation of such reinforcements, but from the SS strengthening of Ti element. However, the strengthening from GND is significantly improved as the reheating temperature rises, due to the thickening of the reinforcement compact shells.

## 4. Conclusions

1. The shells of the core-shell-structured Ti@(Al-Si-Ti)_p_ in the A356 matrix composite are thickened as the reheating temperature rises and a uniform compact shell with 8.45 μm thickness is achieved at 600 °C; then the shells fracture and peel off as the temperature is further elevated. Simultaneously, the firstly formed τ1-phase shells gradually transform into (Al,Si)_3_Ti phase from the shell outside to inside.
2. The tensile properties of the Ti@(Al-Si-Ti)_p_/A356 matrix composite increases as the reheating temperature rises from 590 °C to 600 °C due to the enhanced strengthening role from the thickened compact shells and densified matrix microstructure, and then decrease when the temperature exceeds 600 °C because of the impaired strengthening effect from the fracture and peeling of the compound shells, loosened matrix microstructure, and coarsened primary particles. The composite formed at 600 °C has peak properties, UTS of 371 MPa, YS of 268 MPa, and elongation of 8.3%.
3. Most of the visible Ti@(Al-Si-Ti)_p_ on the fracture surfaces fracture across the Ti core, but some of them occasionally crack around the Ti core, or fracture between the outer fractured shell and the inner core for the composites thixoformed at reheating temperatures higher than 600 °C. For the Ti@(Al-Si-Ti)_p_, cracks first generate within the shells, and then their opening gaps increase accompanied by sever plastic deformation of Ti core. Subsequently, small cracks generate in the Ti core, and they grow up and finally connect with the cracks in the shells, resulting in the fracture of the reinforcement. Cracks that generate in the matrix can also bypass the encountered Ti@(Al-Si-Ti)_p_ along the regions between the outer jagged structures and the inner compact shell, resulting in the ‘debonding’ of Ti@(Al-Si-Ti)_p_/matrix interface.
4. The delayed formation of cracks in the Ti@(Al-Si-Ti)_p_ and their small size are contributed to the ductility improvement of the Ti@(Al-Si-Ti)_p_/A356 composites. The modified MSL model that considers the contributions from GR, GND, TMS, and SS, and introduces a correction factor for the volume fraction of reinforcements is actually suitable for accurately predicting the YS of the composites. The largest contribution is the result of SS strengthening of Ti element, but the strengthening from GND is significantly improved as the reheating temperature rises from 590 °C to 600 °C, due to the thickened shells of the Ti@(Al-Si-Ti)_p_.

## Reference

## Figures and Tables

**Table 1:** Relative densities of the composites thixoformed at different reheating temperatures.

Reheating Temperature, °C	590	600	605	610
Relative density, %	95.45 ± 1.61	97.24 ± 1.63	96.68 ± 1.32	95.13 ± 1.52

**Table 2:** Results from nanoindentation test for different phases in the composites.

Phase	τ1	(Al,Si)_3_Ti	Ti	α-Al
Microhardness, GPa	6.52	4.19	1.33	1.31
Elasticity modulus, GPa	44.67	50.52	24.66	15.71

**Table 3:** Values of the constants used in calculations.

Constant	Value	Ref.	Constant	Value	Ref.
ε	0.002	[30]	S	1	-
α	1	[31]	b	0.22 nm	[23]
G	26 GPa	[32]	∆C	11.7 × 10^−6^ K^−1^	[32]
K	0.1 MN m^−3/2^	[33]	ε’	0.0391	[34]
f	3.61	[4]	E1	216 GPa	[35]
ν	0.33	[32]	E2	112 GPa	[36]

**Table 4:** Measured values of σ_0_, d_Ti_, d_r_ and Δx_f_, and calculated values of C and V_p_ for the composites thixoformed at reheating temperatures of 590 °C and 600 °C.

Reheating Temperatures, °C	σ_0_, MPa	d, µm	d_r_, µm	d_Ti_, µm	Δx_f_, at %	C	V_p_
590	170	37.4	27.1	17.5	0.272	0.69	7.0
600	190	55.2	29.8	12.9	0.393	0.90	9.2

**Table 5:** Calculated values of Δσ_GR_, Δσ_GND_, Δσ_TMS_, and Δσ_SS_ according to Formulas (2)–(5) for the composites thixoformed at reheating temperatures of 590 °C and 600 °C.

Reheating Temperatures, °C	Δσ_GR_, MPa	Δσ_GND_, MPa	Δσ_TMS_, MPa	Δσ_SS_, MPa
590	16.4	9.9	4.7	26.5
600	13.5	17.0	6.1	31.9
